# Application of Digital Image Correlation to Evaluate Strain, Stiffness and Ductility of Full-Scale LVL Beams Strengthened by CFRP

**DOI:** 10.3390/ma16031309

**Published:** 2023-02-03

**Authors:** Michał Marcin Bakalarz, Paweł Piotr Tworzewski

**Affiliations:** 1 Department of Structural Theory and BIM, Kielce University of Technology, Al. Tysiąclecia Państwa Polskiego 7, 25-314 Kielce, Poland; 2 Department of Strength of Building Materials and Structures, Kielce University of Technology, Al. Tysiąclecia Państwa Polskiego 7, 25-314 Kielce, Poland

**Keywords:** CFRP, DIC, EWP, composites, reinforcement, stiffness, strain, strengthening, timber structures

## Abstract

Due to limitations of traditional measuring methods, a necessity of verification of applicability of optical measuring systems in different fields of science is required. The paper presents the application of a non-contact, non-destructive ARAMIS optical system in the analysis of static work of unstrengthened and strengthened laminated veneer lumber beams (LVL) with composite materials, subjected to a four-point bending test. The beams were strengthened with Carbon Fiber Reinforced Polymer (CFRP) sheets and laminates. The sheets were bonded to the external surfaces in three configurations differing in the number of layers applied and the degree of coverage of the side surface. The CFRP laminates were glued into predrilled grooves and applied to the underside of the beams. An adhesive based on epoxy resin was used. The scope of the work includes analysis of the strain distribution, stiffness and ductility. The analysis was performed on the basis of measurements made with an optical measurement system. The strain analysis indicated a change of the distribution of the strain in the compressive zone from linear for the unstrengthened to bilinear for the strengthened beams. The stiffness increase was equal from 14% up to 45% for the application of the CFRP laminates in the grooves and CFRP sheets bonded externally, respectively. Similar improvement was obtained for the ductility.

## 1. Introduction

Wood is one of the oldest construction materials used by humans. It is a renewable raw material with a wide range of beneficial physical and technological properties. The most frequently mentioned advantages of wood and wood-based materials are low weight, the prevalence of the raw material, ease of processing, relatively high tensile and compressive strength and in the case of fire, it burns without emitting harmful substances [1]. However, it is not a perfect material. It has an anisotropic and heterogeneous structure, and its mechanical properties depend on naturally occurring defects such as knots, cracks or twists of grains. External biological, chemical and atmospheric factors can result in the degradation of wooden structures. The prolonged lifetime of a structure, increasing operational loads and changing ambient conditions, accelerates degradation processes. The reasons for defects and deteriorating wooden structures were addressed in manuscripts [2,3,4].

Wooden structures are strengthened to improve or restore the original mechanical properties. A common reinforcement method is the application of additional elements with better mechanical properties that would cooperate with the timber structure. This cooperation is achieved through mechanical, adhesive or mechano-adhesive connections [2]. The issue of strengthening wooden structures is still a significant scientific problem, as confirmed by the number of publications. A selection of them is presented below.

Various configurations and types of reinforcement have been tested over the years [5]. The reinforcement can be placed inside or outside the cross-section in the tensile and/or compressive zone. Currently, elements made out of composite materials reinforced with carbon [6,7,8,9], glass [10,11], basalt [12] and aramid [13] fibers, as well as steel elements [14], are most often used as reinforcement.

Balmori et al. [15] used a uni-directional GFRP sheet as an internal reinforcement of low-grade maritime pine duo beams, achieving an average improvement of 8.37% in bending stiffness and an increase up to 18.45% in the ultimate moment capacity in comparison to the unreinforced beams. Wang et al. [16] studied the structural behavior of small-scale wood beams strengthened externally with Flax, Basalt, E-Glass FRP and their hybrids in three-point bending tests. The FRP type, thickness and the layer arrangement on the flexural behavior of strengthened beams were discussed. They concluded that load-bearing capacity increases with increasing the number of the FRP layers. Ye et al. [17] presented experimental results of the spliced beams with lap joints reinforced with fiber composite materials or steel, indicating better effects of the CFRP sheet reinforcement than that of the CFRP and steel bars due to the better bonding conditions. Similar to the results presented in [16], the strengthening effectiveness increased with the increase in the reinforcement ratio. Rescalvo et al. [18] tested carbon composite materials (laminates and sheets) as passive reinforcement of old timber beams with biological and natural defects. They concluded improvement of the stiffness and bending capacity in comparison with the control specimens. Sokołowski and Kossakowski [19] studied the behavior of solid timber beams strengthened with PBO (p-Phenylene Benzobis Oxazole) mesh. They achieved significant improvement of the destructive force and deflection of the strengthened beams equal to 56% and 77.5% in comparison to the unstrengthened beams. Kawecki and Podgórski [20] conducted experimental, theoretical and numerical studies on glue laminated timber beams strengthened with the CFRP laminates—proving the necessity of including cohesive stiffness of the adhesive layers in the evaluation of the stiffness of the composite wood–CFRP. Rheological relaxation of OSB (Oriented Strand Boards) beams reinforced with CFRP tape was investigated by Socha et al. [21]. Authors achieved a 63% improvement of stiffness and higher carrying capacity for reinforced beams in comparison to beams without reinforcement. In the study by Lukin et al. [22], steel reinforcement of wooden multi-span beams was used, giving a significant improvement of the bearing capacity equal to 175% and reduction in the bearing deformability by 85%.

The studies described above were focused on the analysis of solid and glue laminated timber beams strengthened with composite and steel materials. A limited number of works were devoted to the strengthening of laminated veneer lumber beams with composite materials [6,23,24,25,26,27]. Therefore, the main aim of this article is to create a comprehensive study that includes analysis of the strain, stiffness and ductility of full-scale laminated veneer lumber LVL beams strengthened with CFRP. Different types of strengthening configurations were used, including EBR (Externally Bonded Reinforcement) and NSM (Near Surface Mounted) reinforcement. CFRP sheets and laminates were used as reinforcement and compared. The analysis was conducted on the basis of measurements made by the optical measurement system ARAMIS, verified with the use measurements made with LVDT. The optical system is a non-contact, non-destructive method and because of that it is superior in comparison to traditional methods. Traditional methods are limited by many factors, such as the following: due to interaction with the tested element, they are prone to malfunctions/destruction during the test; measurements are limited to specific areas/points; measurements are mostly made in one-dimension, which can lead to mistakes in analysis. Because of that, it is necessary to verify the applicability of the DIC system for the analysis of the behavior of the bent wood and wood-composite beams. Authors have no knowledge about similar application of the DIC system in terms of scope of analysis, used methodology and subject of analysis.

**Figure 1:**
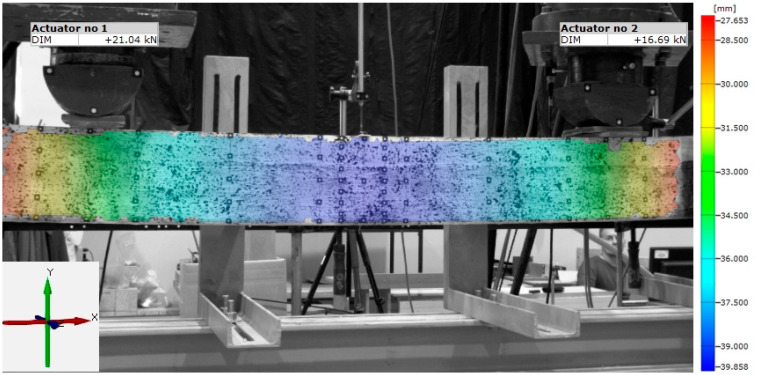
ARAMIS system-map of vertical displacements for beam B1.

Digital Image Correlation enables the registration of the mutual position of individual points on the examined object in space. Capturing and estimating changes in the location of these points in relation to each other is possible by juxtaposing consecutive photos/measurement stages obtained from two or more cameras. The ARAMIS optical measurement system uses Digital Image Correlation (DIC) [28,29]. It enables changes in the location of any point on the recorded surface to be tracked and maps of deformations or displacements to be created. It is made possible by recording the changes in the location of the facets, which means the mesh is applied to the photo of the tested surface. To make it possible, the tested surface is covered with a black and white stochastic pattern, i.e., with no specific tendency. The size of the spots is adjusted to the size of the examined area and the resolution of the cameras. Figure 1 shows an exemplary map of vertical displacements (Y direction) for B1 beam. Optical measurement systems more and more often supplement or even replace other devices. Their great advantage is the so-called non-contact measurements, a wide range of measurement possibilities and the simultaneous obtaining of photographic documentation [29,30].

The DIC measurement technique is also economical. There is no risk of damaging the components of the apparatus and the only expenditure is related to the preparation of patterns. It can be considered that this method, and more precisely 2D-digital image correlation (2D-DIC), was first used by Sutton et al. in 1983 [31]. Later, this method was developed into 3D-DIC methods through the use of multiple cameras. The history of the evolution of this method is presented in more detail in [32].

As mentioned earlier, the DIC method is mainly used for complex strain and deflection measurements. Jeong et al. [33] analyzed the bearing properties of the differently oriented glulam. For this purpose, strain distributions near the bearing area of the specimens were obtained using Digital Image Correlation. Navaratnam et al. [34] used DIC analysis to evaluate the failure modes, crack growth rate and inclined angle of shear cracks for Australian radiata pine CLT panels. Liu et al. [35] used this method to determine the dynamic impact–resistance properties of wood beams reinforced with carbon fiber reinforced polymers (CFRPs). Lima et al. [36] determined the elasticity modulus in Eucalyptus grandis and Pinus oocarpa wood specimens using the PIV and DIC techniques. Accuracy and applicability of these two methods, in relation to the conventional testing technique, were verified. The analysis showed that, when comparing the three techniques, the results of the elasticity modulus were not different (*p* < 0.05). Kawecki [37] used strain maps obtained for wood–CFRP beams using the Digital Image Correlation (DIC) method to validate the developed FEM model. Tang et al. [38] proposed a new crack backbone refinement algorithm and width-measurement scheme. The practical application of the method, based on analysis of cracks of a reservoir dam, was presented.

Simultaneously, researchers conduct material studies to improve composites from economical, ecological, technological or mechanical points of view. Verma et al. [39], described a methodology to fabricate and characterize the carbonaceous filler modified epoxy resin. As a filler, a nanocarbon black obtained from the pyrolysis of waste tires was used. The filler content on physical, mechanical, electrical and thermal properties was analyzed. In paper [40], the mechanical analysis and microstructure investigation of chicken feather fiber with carbon residuum were presented. Improvement of hybrid composite in comparison to neat epoxy resin was achieved.

## 2. Materials and Methods

### 2.1. Materials

#### 2.1.1. Laminated Veneer Lumber

**Figure 2:**
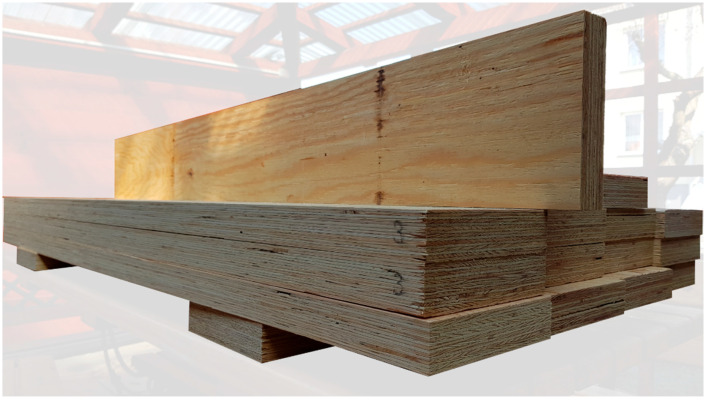
View of the LVL beams.

Tests were carried out on full-size beams made of laminated veneer lumber with nominal dimensions of 45 × 200 × 3400 mm. Each element consisted of 15 approximately 3 mm thick layers of pine or spruce veneers. Beams were tested in an edgewise condition. The humidity of the veneer was approximately 14%. A view of the LVL beams is presented in Figure 2.

Selected mechanical and physical properties of laminated veneer are shown in Table 1.

#### 2.1.2. Carbon Fiber Reinforced Polymer

**Figure 3:**
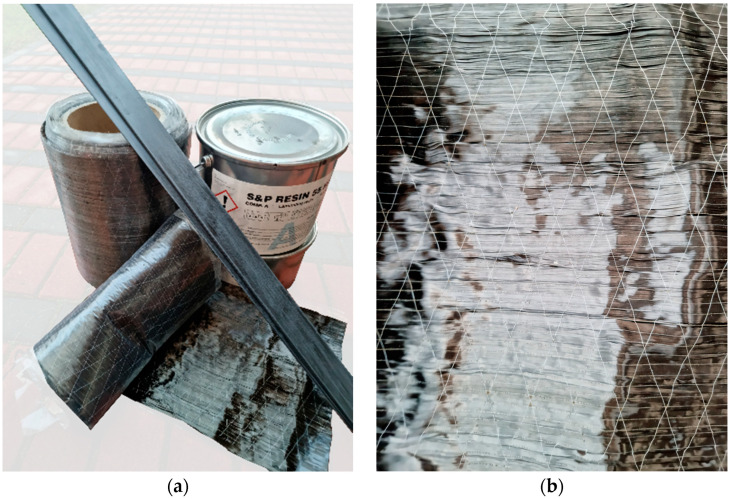
Strengthening system: (**a**) components of composite reinforcement; (**b**) CFRP sheet.

Uni-directionally reinforced CFRP sheets and laminates were used to reinforce the LVL beams (Figure 3).

The sheets were delivered in rolls with a width of 300 mm. Dimensions of the cross-section of the laminates glued into the grooves were 1.4 × 20 mm, and the laminates glued to the outer surface were 1.4 × 43 mm. The length of reinforcement was 2800 mm. The selected mechanical and physical properties of the CFRP reinforcement are shown in Table 2.

#### 2.1.3. Epoxy Resin

Two types of adhesives were used for the application of the FRP reinforcement. Both of them are based on epoxy resin. S&P Resin 55 HP adhesive was used for gluing sheets and bonding laminates into grooves. S&P Resin 220 adhesive was used for gluing the CFRP laminate to the bottom surface. Selected physical and mechanical properties of the adhesives are exhibited in Table 3.

#### 2.1.4. Preparation for Application of Reinforcement

In this section, selected aspects of the application of the reinforcement procedure regarding the preparation of the LVL and composite are presented.

**Figure 4:**
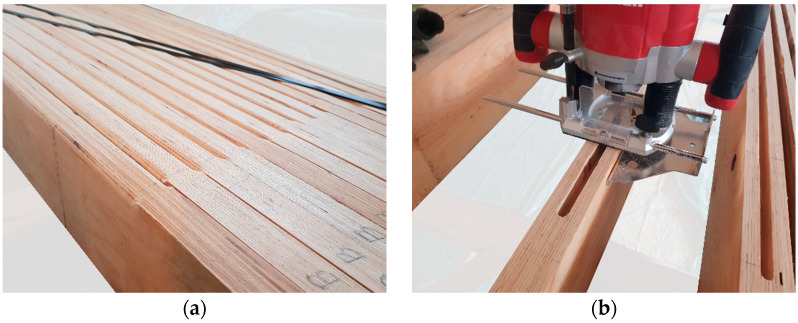
View of prepared LVL beams: (**a**) with rounded corners; (**b**) with groove.

Preparation of the LVL beams for application reinforcement included (Figure 4) the following: marking the position of the FRP reinforcement on the LVL surface; grinding the side surface and rounding corners covered by the CFRP sheet; drilling the grooves for application of the CFRP inserts; and finally, cleaning the surface from dust or wood residues.

**Figure 5:**
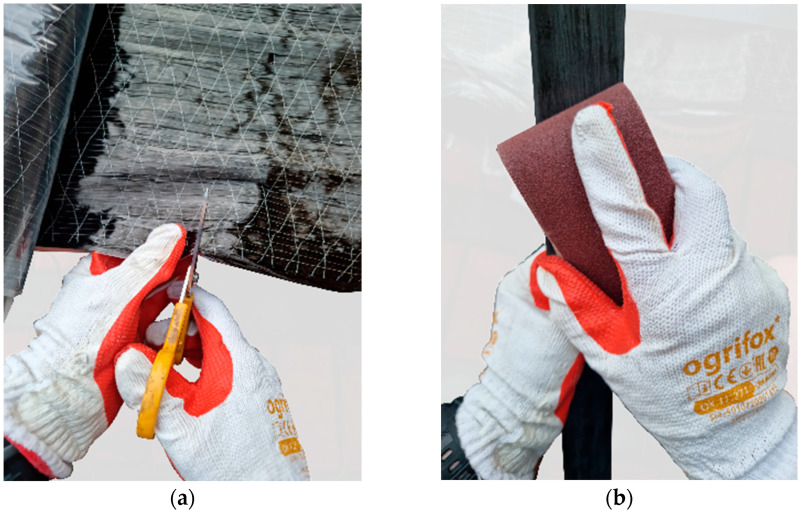
Preparation of CFRP reinforcement: (**a**) cutting CFRP sheet; (**b**) roughing CFRP laminate surface.

Prior to the application, the CFRP composites were cut and cleaned. The sheets were cut to the assumed dimensions using scissors, and the laminates were cut with a circular saw. For cleaning, cloth and sandpaper (for laminates) were used. The preparation of the CFRP sheets and laminates is shown in Figure 5.

### 2.2. Method

#### 2.2.1. Bending Test

**Figure 6:**
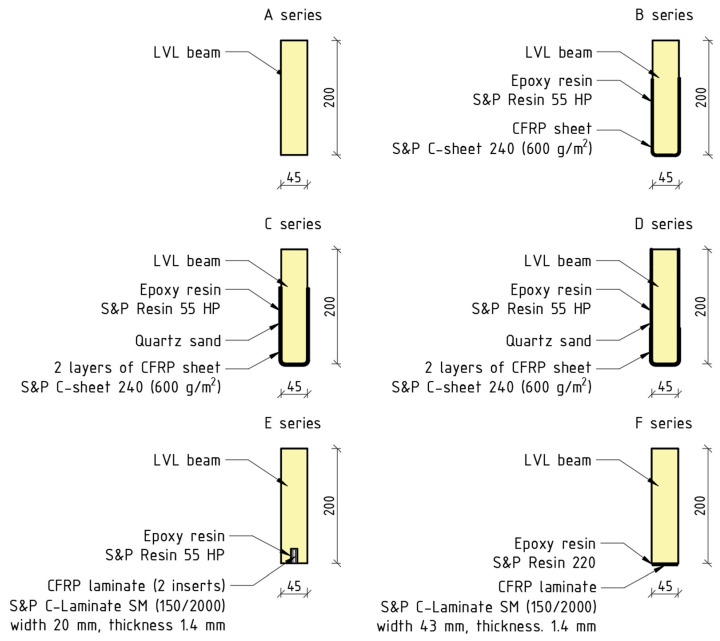
Strengthening configurations: A—unstrengthened beams; B—strengthened with one layer of CFRP sheet; C—beams strengthened with two layers of CFRP sheet bonded one on another; D—beams strengthened with two layers of CFRP sheet covering entire side surface; E—beams strengthened with two CFRP laminates bonded into predrilled grooves; F—beams strengthened with CFRP laminated bonded to bottom surface.

Tests were conducted at the Kielce University of Technology. Bending tests were conducted on simply supported beams according to the guidelines enlisted in the standards [46,47]. The scope of the tests involved the preparation of six test series (Figure 6): A—reference beams; B—beams reinforced with one layer of the CFRP sheet; C—beams reinforced with two layers of the CFRP sheet bonded in the tensile zone; D—beams reinforced with two layers of the CFRP sheet covering the entire side surfaces; E—beams reinforced with the CFRP laminates bonded into the grooves (two inserts in the groove); F—beams reinforced with a laminate bonded to the external surface. Each series consists of five beams. The beam testing procedure is presented in detail in [23,24,25].

**Figure 7:**
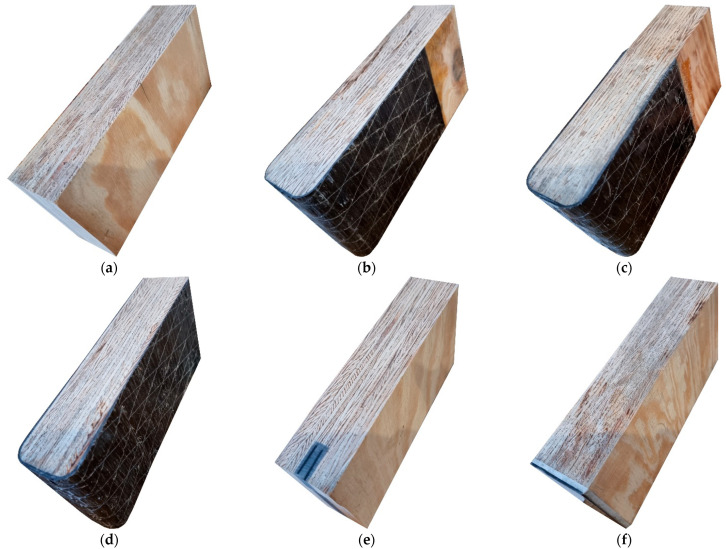
Views of cross-sections of tested beams for series: (**a**) A; (**b**) B; (**c**) C; (**d**) D; (**e**) E; (**f**) F.

The examples of the cross-sections for each test series are shown in Figure 7. The cross-sections were cut out after the bending test.

**Figure 8:**
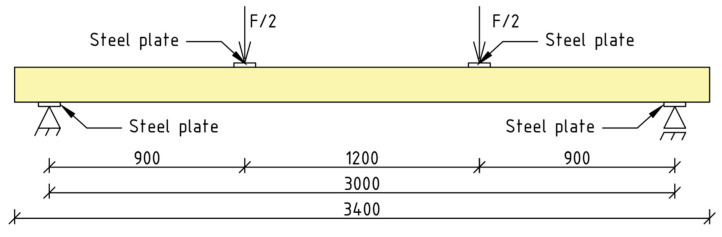
Scheme of static test setup.

The beams were symmetrically loaded with two concentrated forces. The span of the beams in the axis of supports was 3000 mm. The distance between the concentrated forces was 1200 mm. The distance from the support axis to the axis of the nearest concentrated force was 900 mm. The load was controlled by the shift speed of the loading pressures—a speed of 7 mm/min was applied. The initial load was 0.3 kN in each actuator. Steel guide plates of 100 mm width were applied in the point of applying the concentrated force and on the supports. The static diagram of the test stand is shown in Figure 8.

#### 2.2.2. Digital Image Correlation System

**Figure 9:**
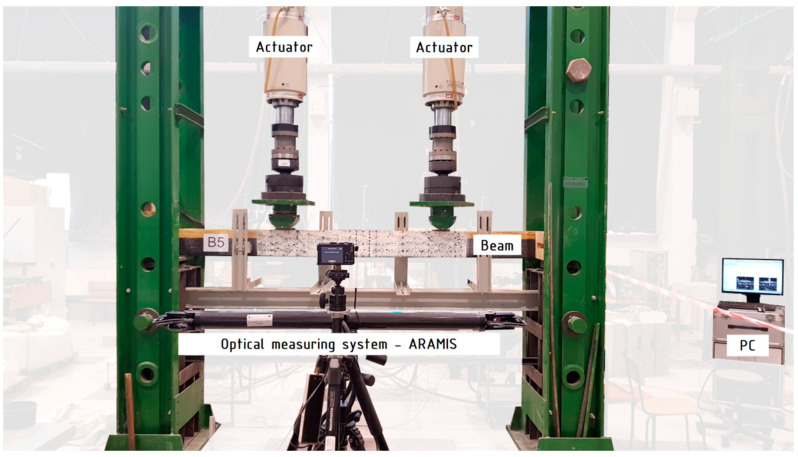
View of the static test setup.

The ARAMIS non-contact optical system was used to measure the displacements and deformations of the lateral surface. The system consisted of a central unit in which the data obtained from experimental research were stored and analyzed, and a set of cameras placed on a tripod with a large measuring arm. Baumer TXG50 cameras with a resolution of 2448 × 2050 pixels and a Schneider Kreuznach Cinegon 1.4/12-0906 lens were used. The distance between the cameras on the tripod was 1200 mm. The width of the tested surface depends on the distance to the observer and the assumed measurement accuracy. The load value was recorded as a voltage signal using the Labtronic controller and RS BasTest software. A standard arrangement of facets of 15 × 15 pixels with an overlap of 2 pixels was adopted. The view of the test stand is shown in Figure 9.

**Figure 10:**
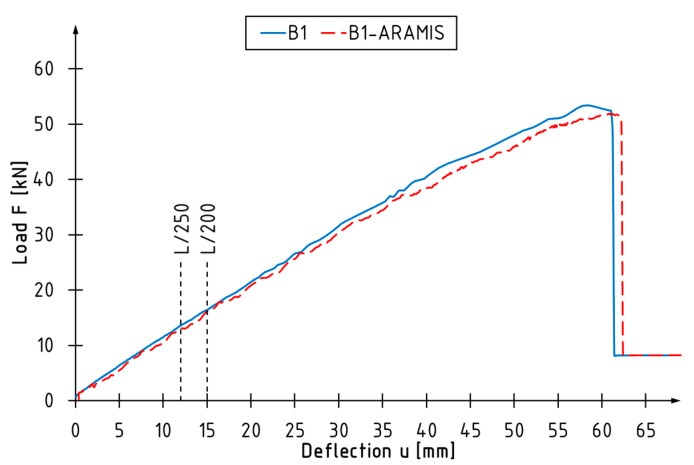
Comparison of midspan deflection measured using DIC and LVDT system in the function of load.

The data using the DIC system were collected from the beginning of the test till the failure. The accuracy of optical measurements was verified with the use of the midspan deflection values recorded with LVDT—good agreement was achieved (Figure 10). The offset of the curves was caused by the methodology of recording the deflection of the beam in each system. The DIC records the displacement in three-dimensional space, as with a deflection of the beam a vertical component is assumed. LVDT records the displacement of the specific point at which it is applied—in that way, the lateral displacement of the beam during bending is not included.

**Figure 11:**
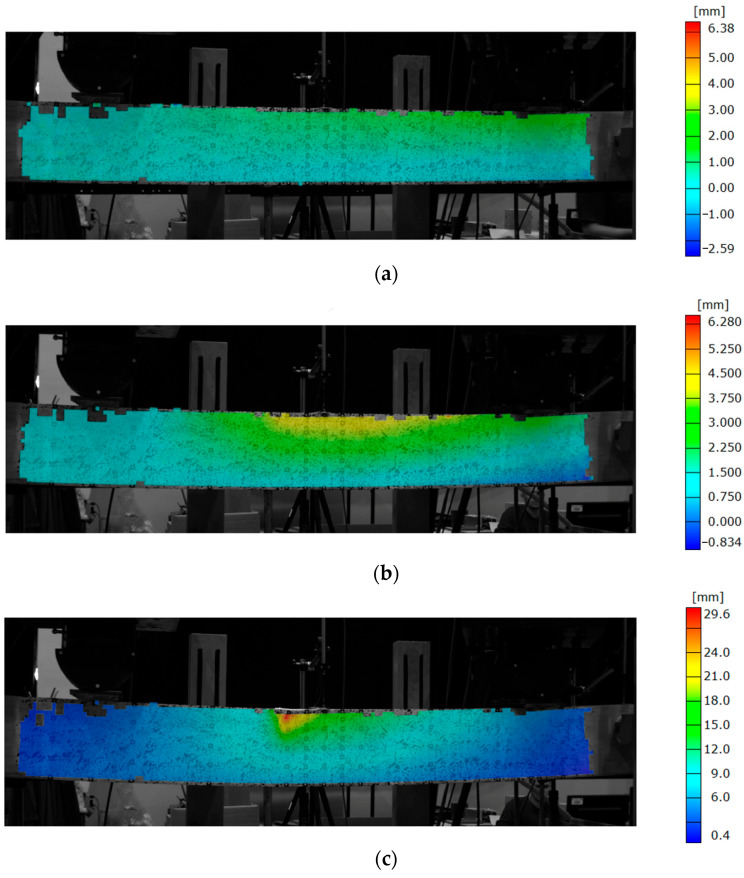
Evolution of lateral displacement of beam, at: (**a**) load equal to 0.5 Fmax; (**b**) failure load Fmax; (**c**) failure propagation.

To better visualize the possible error in the measurement of the deflection using the traditional methods, maps of lateral displacement for the beam are shown in Figure 11. Due to testing relatively slender beams, increasing the load caused twisting of the beam. The highest horizontal displacements were recorded at the top of the beam, where the LVDT system was applied.

The measurement uncertainty verification for the ARAMIS system was based on two stages: stage 0 and stage 1, which were recorded for an unloaded beam, and the displacement values were measured at 1370 points (Table 4). The Gaussian distribution was used to calculate the measurement uncertainty due to the large number of measurements. In order to obtain almost absolute certainty (*p* = 0.997) that the real value is within the range defined by the measurement uncertainty, the rule was used, i.e., the triple value of the standard deviation was applied. The results of the calculations are presented in Table 4. Based on the calculations, it was determined that the measurement uncertainty for the configuration of the ARAMIS system used in the tests is ±0.010 mm.

#### 2.2.3. Strains

**Figure 12:**
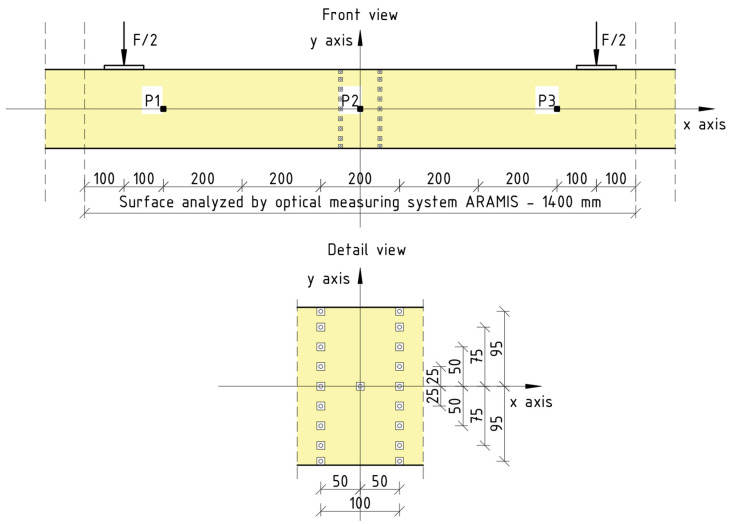
Arrangement of measuring points—DIC reference points.

The strains of the beams in a middle profile were estimated based on the changes in the displacements of the measurement points in the *xy* plane. The measurements were taken on nine levels at the height of the cross-section. The vertical distance between the points was 25 mm, and the horizontal was 100 mm. A diagram of the distribution of measurement points (DIC reference points) in the middle part of the beam is shown in Figure 12.

#### 2.2.4. Ductility

The bending ductility can be defined as the capability to withstand plastic deformations by maintaining the appropriate level of load-bearing capacity. In the presented tests, the bending ductility of the beams was evaluated using the indices based on the relationships between the deflection values in the characteristic points *D*, *µ_d_*, *µ*_Δ_ and a measure using quotients of the absorbed *µ_E_* energy.

The deflection index *D*, described by Borri et al. [48], is evaluated according to Equation (1):

(1) $$D=\frac{u_{s}}{u_{un}}$$

where:

*u_s_*—the deflection at the maximum load of the strengthened beam,
*u_un_*—the deflection at the maximum load of the unstrengthened beam.

The ductility index *µ*_Δ_ is defined as the quotient of the deflection at the ultimate load to the deflection at the yield point according to the Formula (2) [49]:

(2) $$\mu_{\Delta }=\frac{u_{u}}{u_{y}}$$

where:

*u_u_*—the deflection at the ultimate load,
*u_y_*—the deflection at the yield point.

In paper [50], the ductility index, based on the deflection value at the proportionality limit, was described by Formula (3):

(3) $$\mu_{d}=\frac{u_{u}}{u_{e}}$$

where:

*u_e_*—the deflection at the elastic limit.

The ductility index *µ_E_* expressed as the quotient of the total and elastic energy is evaluated from Formula (4) [51]:

(4) $$\mu_{E}=\frac{1}{2}\cdot \left(\frac{W_{tot}}{W_{e}}+1\right)$$

where:

*W_e_*—the elastic energy (fraction of total), the area under the load-deflection curve up to elastic limit;
*W_tot_*—the total energy, the area under the load-deflection curve up to failure.

#### 2.2.5. Stiffness

The bending stiffness of the unreinforced and reinforced laminated veneer lumber beams was demonstrated by the local stiffness coefficient *k_l_* and global *k_g_* stiffness coefficient, as well as the local *E_m,l_* and global *E_m,g_* stiffness modulus. The values of the analyzed parameters were estimated for the linearly elastic part of the load-deflection diagram.

##### Stiffness Coefficients

Local stiffness coefficient *k_l_* was evaluated on the basis of the displacement of the P2 point in reference to the line drawn through the P1 and P3 points, and the corresponding load *F* according to Equation (5) (based on [52]):

(5) $${F=k}_{l}\cdot \;f\;\to k_{l}=\frac{F}{f}.$$

where:

*F*—the loading force,
*f*—the local deflection.

The global stiffness coefficient *k_g_* was evaluated on the basis of the deflection measured in the middle of the beam *u* and the corresponding load *F*, according to Equation (6):

(6) $${F=k}_{g}\cdot \;u\;\to k_{g}=\frac{F}{u}.$$

where:

*u*—the deflection.

##### Modulus of Elasticity

The local modulus of the elasticity at the bending *E_m,l_* was evaluated from Formula (7) [46]:

(7) $$E_{m,l}=\frac{a\;\cdot l_{1}^{2}\cdot \Delta F}{16\cdot I\;\cdot \Delta u}$$

where:

*a*—the distance between the concentrated load and the nearest support axis,
*l*_1_—the gauge length for the determination of the modulus of elasticity,
*I*—the second moment of inertia,
Δ*F*—the increment of load,
Δ*u*—the increment of the deflection corresponding to the increment of the load.

The global modulus of elasticity at the bending *E_m,g_* was evaluated from Formula (8) [46]:

(8) $$E_{m,g}=\frac{3\;\cdot \;a\;\cdot {\;l}^{2}-4\;\cdot {\;a}^{3}}{2\;\cdot \;b\;\cdot {\;h}^{3}\left(2\;\cdot \;\frac{\Delta u}{\Delta F}-\frac{6\;\cdot \;a}{5\;\cdot \;G\;\cdot \;b\;\cdot \;h}\right)}$$

where:

*l*—the span in bending,
*b, h*—the cross-section dimensions,
G—the shear modulus.

## 3. Results and Discussion

To verify the improvement of the mechanical properties of the LVL beams reinforced with carbon composites, the test results were discussed in the field of the strains of the middle profile, the increase in the stiffness and the bending ductility.

### 3.1. Strain Analysis

**Figure 13:**
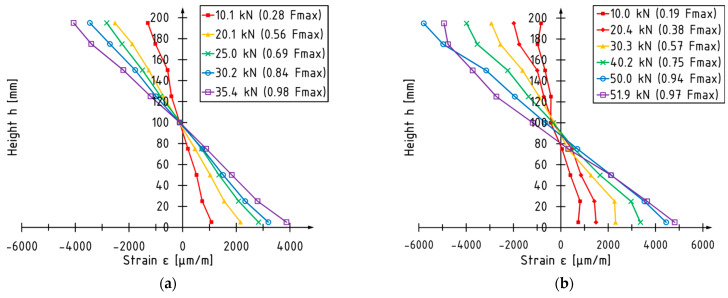

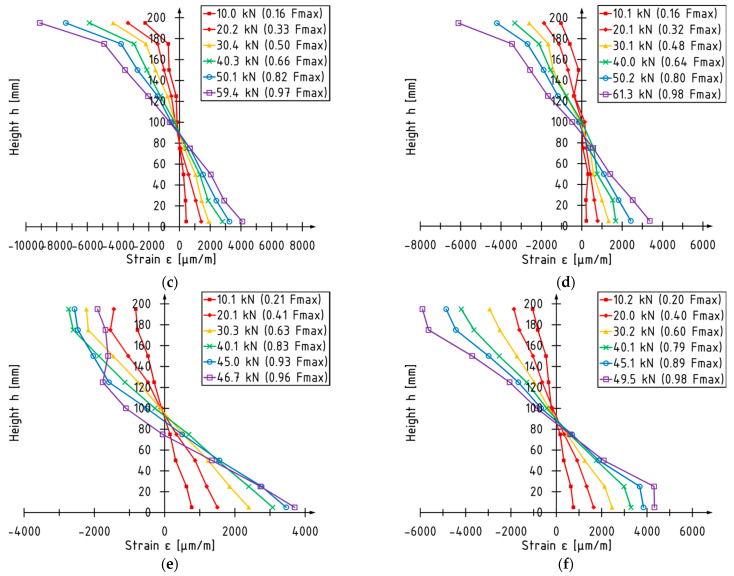
Strain profiles of selected tested beams: (**a**) A5; (**b**) B1; (**c**) C4; (**d**) D4; (**e**) E2; (**f**) F5.

The strain profiles, recorded in the middle section, for the selected tested beams from each series are shown in Figure 13. The distribution of the strains along the depth of the cross-section depends on several factors including the following: the presence of defects in the veneers including voids after knots; the type of reinforcement; and the location of the reinforcements. Furthermore, the distribution is dependent on the measuring location—the position of the section in regard to the position of failure. Therefore, the strain measurements for the entire side surface should be superior for describing the behavior of the bent beams in comparison to the point (local) measurements.

The linear strain distribution in the compression and tension zone was obtained for all unstrengthened beams. In the case of the LVL beams reinforced with the CFRP sheets and CFRP laminates glued into grooves, the distribution of strains in the compressive zone is non-linear and linear in the tensile zone. The non-linear part consists of a linear and parabolic shape. For the C4 and D4 beams (strengthened with two layers of the CFRP sheet), there was a significant increase in the strains measured at the level of the outermost compressed fibers—this was due to the failure that occurred outside the deformation measurement zone. In the case of an LVL beam reinforced with a CFRP laminate glued to the bottom surface of the beam, apart from the plasticization of the compressed zone (which was the failure mode of beam), the tensile zone was also plasticized.

The position of the neutral axis of the unstrengthened beams is located at the the geometric centroid of the cross-section. The position of the neutral axis of the beams reinforced with the composite materials is shifted toward the outermost tension fibers. The shift value is not constant throughout the test—it depends on the failure mode and load value.

The maximum value of the measured strains in the tensile zone for the unstrengthened and strengthened beams is approximately the same and equals 4000 µm/m. For unstrengthened elements, the maximum strain value in the compressive zone is equal to the strain in the tensile zone. For the strengthened elements, the strains measured in the compression zone are usually higher than the strains in the tensile zone. These values differ more than twice.

Besides the analysis of the strains in specific sections, the optical measurements system allows for presenting maps for the entire surface. This feature is especially useful where the complex failure of the beam occurs with several initiation points. It can be also used for presenting the failure modes.

**Figure 14:**
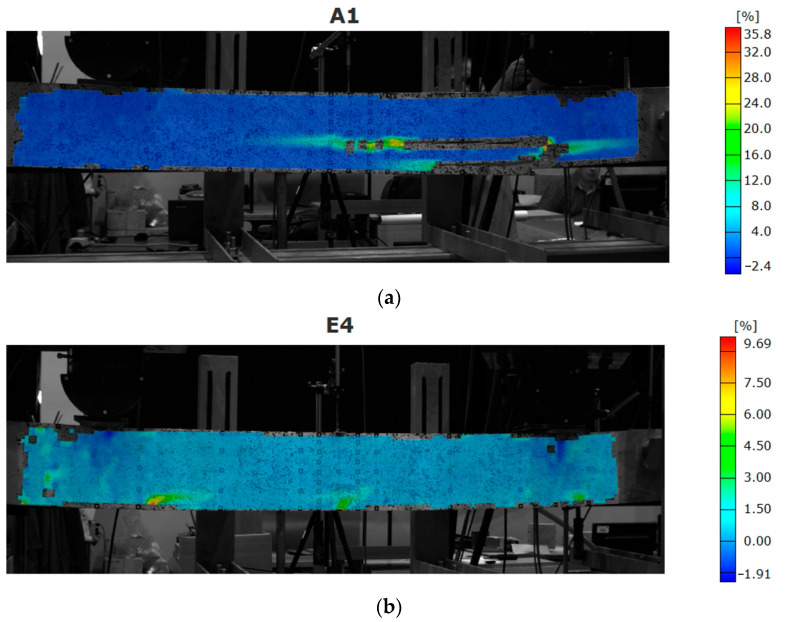
Strain maps for beam: (**a**) A1; (**b**) E4 (beam strengthened with CFRP laminates bonded inside grooves).

The unreinforced beams and the weakly reinforced LVL beams failed due to exceeding the tensile strength of the LVL (Figure 14). For beam E4, several points of initiation failure were recorded along the bottom edge. These points indicate the place where the debonding of the CFRP occurred.

**Figure 15:**
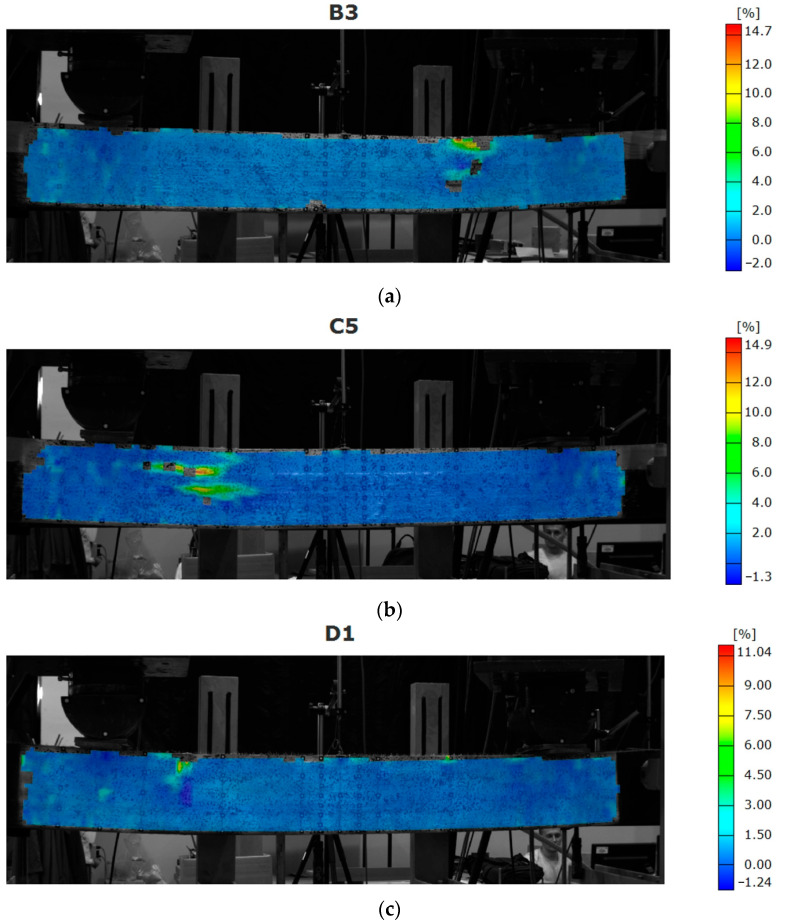
Map of strain distribution for beams strengthened with CFRP sheets: (**a**) B3 (one layer of CFRP sheet); (**b**) C5 (two layers of CFRP sheet bonded one on another); (**c**) D1 (two layers of CFRP sheet covering entire side surface).

Typical failure of the beams strengthened with the CFRP sheets involved failure of the LVL in the compressive zone (Figure 15). The rupture of the CFRP sheet occurred when only one layer was applied. For the beams strengthened with two layers, the CFRP sheet failed in the compressive zone due to the displacement of the veneers.

**Figure 16:**
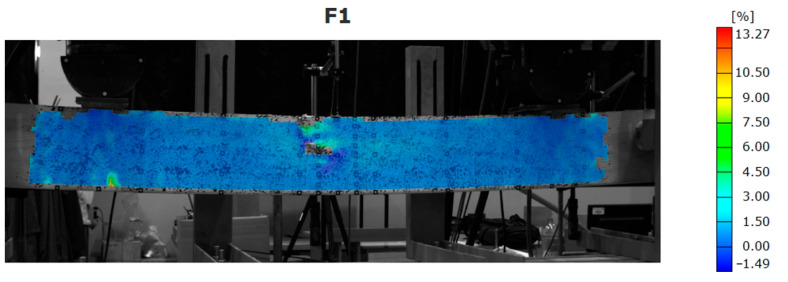
Map of strain distribution for beam strengthened with CFRP laminate bonded to the bottom surface.

The beams strengthened with the CFRP laminate failed due to exceeding the compressive strength parallel to the grain of the laminated veneer lumber with the debonding of the CFRP laminate (Figure 16). The debonding started at the end of the laminate and propagated usually to the middle of the beam.

Although the recording strains for the entire surface is convenient, the large deformations of the analyzed surface can generate the blank spaces on them. That phenomenon is visible for places where failure occurred—mainly due to the displacement of the veneers (Figure 14, Figure 15 and Figure 16).

### 3.2. Ductility

**Figure 17:**
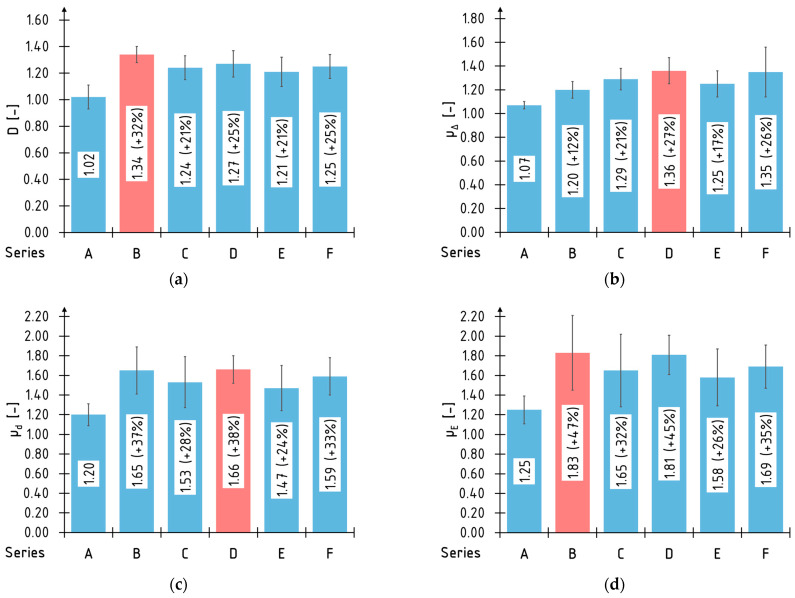
Average values and standard deviation of ductility indexes: (**a**) *D*; (**b**) *μ*_Δ_; (**c**) *μ_d_*; (**d**) *μ_E_*.

The average values of the estimated ductility indices for all the tested series are shown in Figure 17. The series with the maximum percentage increase in comparison with the A series were marked with the red color. It is impossible to indicate unequivocally which of the applied ductility indices is the best to describe this phenomenon. However, it has to be considered that the results differ much depending on which ductility index is applied. For example, the B series beams are characterized by the highest increase in ductility according to the *D* and *µ_E_* indices; at the same time, these beams ranked worst when applying the *µ*_Δ_ index.

The highest percentage increases were obtained by applying the index based on *µ_E_* energy absorption, amounting to, respectively: above 40% for the B and D series beams; above 30% for the C and F series beams; and above 20% for the E series. The smallest ones applied the *µ*_Δ_ index. Parameter *D* has the most homogeneous (flattened results) values—it is related to the similar values of the deflection at the failure of the beams.

The differences in the above mentioned values were caused by the differences in the formulas used in their evaluation. For example, the deflection based indices (*D*, *µ*_Δ_ and *μ_d_*) used the deflection values, which were read for specific points called “characteristic” and were recorded during the test. At the same time, they ignored the load value corresponding to the deflection. It should be mentioned also that sometimes evaluating the location of these characteristic points can be problematic. The energy based index *μ_E_* took into account that relation by evaluating the area under the load-deflection curve.

The key feature limiting the ductile behavior of the tested laminated veneer lumber beams was its composite structure—the beam structure consisted of relatively thick veneers bonded together. The main failure mode of the strengthened beams (with enough reinforcement to prevent failure in the tensile zone) was the loss of stability of the compressed veneers. Under compression of the veneers, the buckling and debonding of the adjacent layers occurs.

### 3.3. Stiffness

#### 3.3.1. Local and Global Stiffness Coefficients

**Figure 18:**
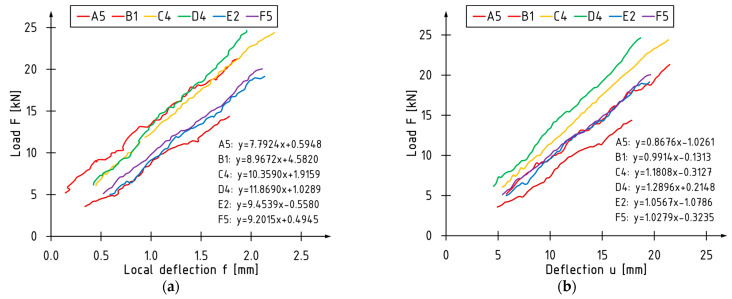
Relationship in range from 0.1 to 0.4 *F_max_* for: (**a**) load—local deflection; (**b**) load global deflection.

The load versus the deflection curves used for the evaluation of the local stiffness coefficient are shown on Figure 18a. Similarly, Figure 18b shows the load-deflection curves for the global stiffness coefficient. The heterogeneous waveform of the curves results from the frequency of the pictures being taken by the optical system. To avoid errors caused by the shape of the curve, the direction factor of each curve was used in the subsequent calculations of the local and global stiffness coefficients. The bending stiffness was evaluated in the of 0.1 to 0.4 *Fmax*, which describes the linear relationship between the load and deflection. The graph shows slight differences in the slope of the B, E and F series beams. The visibly greater slope is observed in the C and D series elements. The curve of the A series beam has the smallest slope.

**Figure 19:**
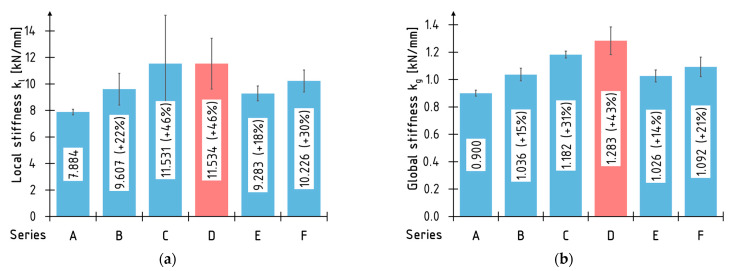
Average values of stiffness coefficients: (**a**) local k*_l_*; (**b**) global *k_g_*.

The average values of the global and local bending stiffness coefficient and their percentage increases relative to the A series were demonstrated in Figure 19. The highest percentage increase in the *k_l_* and *k_g_* stiffness coefficient was recorded for the beams reinforced with the two layers of CFRP sheet—C and D series. Comparable (lowest percentage increases) stiffness coefficient values were obtained for the beams reinforced with one layer of the CFRP sheet (B series) and the strips glued into the grooves (E series). The application of carbon laminate on the bottom surface turned out to be a middle solution.

#### 3.3.2. Global and Local Modulus of Elasticity

**Figure 20:**
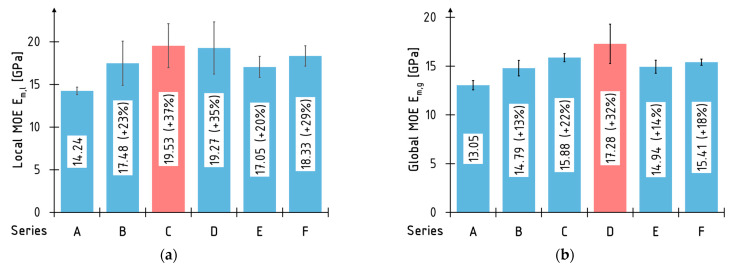
Average values of modulus of elasticity: (**a**) local *E_m,l_*; (**b**) global *E_m,g_*.

Figure 20 describes the average global and local elastic modulus values for each tested series and the percentage increase for the beams strengthened with the composite materials. The highest average increase in the local and global elastic modulus in bending were estimated for the beams strengthened with two layers of the CFRP sheet (series C and D). The lowest increases were recorded for the series E beams.

The values of the local modulus of elasticity and the percentage increases for the strengthened beams are significantly higher than the corresponding values estimated for the global modulus of the elasticity. These differences are caused by way of the curvature of the beam as it is analyzed during bending. For the local modulus of elasticity, the curve is defined by the three points lying between the concentrated forces—point in the middle of beam and the two points shifted 500 mm from the middle in each direction along the beam axis. For the global modulus of elasticity, the curve is defined by the point in the middle and on the supports.

It should be noted that in the evaluation of the local and global modulus of the elasticity, the solid cross-section characteristics were used.

## 4. Conclusions

This manuscript describes the results of the strain, ductility and stiffness analyses of the unstrengthened and strengthened beams with the CFRP composite laminated veneer lumber beams under static load. The analysis was based on measurements made with the non-contact ARAMIS optical measuring system. A total of 30 beams, 5 unstrengthened and 25 strengthened with the CFRP sheets or laminates, were subjected to four-point bend tests. Five configurations of beam reinforcements were compared. The most important conclusions from the conducted research are presented below:

- The ARAMIS optical system can be used for non-destructive examination non-interfering with the side surface of unstrengthened and strengthened wooden elements. The applied measurement system allows for making a wide range of deformations, strains, stiffness and ductility analyses.
- The reinforcement of beams using two layers of the CFRP sheets is the most effective method of beam reinforcement among the discussed strengthening configurations, given the increase in stiffness and bending ductility. Better results can also be achieved when the CFRP sheet covers the entire side surfaces.
- Better mechanical parameters are observed for the beams strengthened with the CFRP laminates glued to the bottom surface rather than glued into the predrilled grooves.
- Strain profiles analysis indicated changes in the distribution of the strain in the compressive zone from the linear for the unstrengthened to the bilinear (consisted of linear and parabolic part) for the strengthened LVL beams.
- Distribution of strains along the depth of the cross-section depends on the presence of defects in the LVL, the type of reinforcement and its location. Reading of the strains depends on the distance between the section where the strains are measured and the section where the failure occurs.
- Higher values of the stiffness coefficient and the modulus of elasticity were obtained for the local in comparison to the global values. It is caused by the way of evaluating the curvature of the beams during bending in each method.

The ductility and stiffness analysis of the LVL beams on a laboratory scale and strengthened with aramid, glass, and carbon sheets with high strength and ultra-high modulus of elasticity were described in [26].

## Figures and Tables

**Table 1:** Mechanical and physical parameters of laminated veneer lumber (exposed by manufacturer) [41].

Parameter	Value
Bending strength, edge wise [MPa]	44
Bending strength, flat wise [MPa]	50
Tension strength (parallel to grain) (for 3000 mm sample) [MPa]	36
Tension strength (perpendicular to grain) [MPa]	0.9
Compression strength (parallel to grain) [MPa]	40
Compression strength (perpendicular to grain) [MPa]	7.5
Shear strength (parallel to grain) [MPa]	4.6
Modulus of elasticity [GPa]	14
Shear modulus [MPa]	600
Density [kg/m^3^]	550

**Table 2:** Mechanical and physical parameters of composite reinforcement (exposed by manufacturer) [42,43].

Parameter	S&P C-Sheet 240	S&P C-Laminate
Modulus of elasticity [GPa]	265	170
Tensile strength [Mpa]	5100	2800
Density [kg/m^3^]	1800	1600
Elongation at rupture [%]	1.7–1.9	1.6
Thickness [mm]	0.333	1.4

**Table 3:** Mechanical and physical parameters of adhesive (exposed by manufacturer) [44,45].

Parameter	S&P Resin 55 HP	S&P Resin 220
Modulus of Elasticity [Gpa]	3.2	7.1
Compression strength [Mpa]	100	70
Density [kg/m^3^]	1200–1300	1700–1800

**Table 4:** Measurements uncertainty using ARAMIS.

Number of Measurements	Average Value [mm]	Standard Deviation [mm]	Measurement Uncertainty [mm]
*n*	$\bar{x}=\frac{1}{n}\overset{n}{\underset{k=1}{\sum }}x_{k}$	$S_{\bar{x}}=\sqrt{\frac{\sum_{k=1}^{n}{\left(x_{k}-\bar{x}\right)}^{2}}{n\left(n-1\right)}}$	$\Delta x=3S_{\bar{x}}$
1370	0.0039	0.0034	0.010

## Data Availability

Data sharing is not applicable to this article.
